# The use of pedicled buccal fat pad combined with sequestrectomy in bisphosphonate-related osteonecrosis of the maxilla

**DOI:** 10.4317/medoral.17422

**Published:** 2011-12-06

**Authors:** Lorena Gallego, Luis Junquera, Alejandro Pelaz, Josué Hernando, Joaquim Megías

**Affiliations:** 1 DDS,MD,PhD, Staff Surgeon, Department of Oral and Maxillofacial Surgery, Cabueñes Hospital, Gijón, Spain; 2 DDS,MD,PhD, Adjunct Professor, University of Oviedo Dental School, Spain. Staff Surgeon, Department of Oral and Maxillofacial Surgery, Central University Hospital, Oviedo, Spain; 3 DDS, MD, Attending, Department of Oral and Maxillofacial Surgery, Central University Hospital, Oviedo, Spain; 4 MD, Attending, Department of Oral and Maxillofacial Surgery, Central University Hospital, Oviedo, Spain

## Abstract

The use of pedicled buccal fat pad flap (BFP) has proved of value for the closure of oroantral and oronasal communications and is a well-established tool in oral and maxillofacial surgery. Otherwise, the perceived limitations of surgical therapy for bisphosphonate-related osteonecrosis of the jaws (BRONJ) have been widely discussed, and recommendations have largely been made to offer aggressive surgery only to stage 3 patients refractary to conservative management. Oroantral communication may be a common complication after sequestrectomy and bone debridement in upper maxillary BRONJ. We report a case series of stage 3 recalcitrant maxillary BRONJ surgically treated with extensive sequestrectomy and first reconstruction using pedicled BFP. All the cases presented an uneventful postoperative healing was uneventful without dehiscence, infection, necrosis or oroantral communication. We postulate that managing initially the site with BFP and primary closure may ensure a sufficient blood supply and adequate protection for an effective bone-healing response to occur. This technique may represent a mechanic protection and an abundant source of adipose-derived adult stem cells after debridement in upper maxillary BRONJ. We evaluate in this work results, advantages and indications of this technique.

** Key words:** Buccal fat pad flap, bisphosphonate-related osteonecrosis of the jaws, oroantral communications, sequestrectomy.

## Introduction

A pedicled buccal fat pad flap (BFP) was first described by Egyedi in 1977 for the closure of oroantral (OACs) and oronasal communications secondary to oncologic resections (1). During the past 3 decades, it has proved of value for the closure of OACs and is a well-established tool in oral and maxillofacial surgery. It has been used as a pedicled graft in facial augmentation procedures, for the repair of persistent oroantral fistulas after dental extractions, and in the treatment of oral submucous fibrosis (2,3).

Bisphosphonate-related osteonecrosis of the jaws (BRONJ) is an enigmatic pathologic entity that was described in the scientific literature initially in 2003 by Marx et al. (4) A clinical stage classification has been proposed, based on clinical symptoms (mainly pain) and the presence of lesions and complications such as jaw fractures, bone sequestrum and skin fistulas by Ruggiero et al. (5) Treatment strategies varies depending stage of BRONJ, although literature regarding the treatment of an established disease (stage 3) is not conclusive. American Association of Oral and Maxillofacial Surgeons (AAOMS) purpose conservative debridement, including resection, combined with antibiotic therapy in these patients (6). Recently, some authors describe high success rates developing aggressive resections (7,8). 

OACs may be a common complication after sequestrectomy and bone debridement in upper maxillary BRONJ, avoiding spontaneous healing and results in chronic fistulas. We evaluate the use of BFP for first reconstruction of refractary maxillary BRONJ surgically treated with extensive sequestrectomy. The effectiveness of this technique for OAC prevention, advantages and indications are discussed.

## Case Series

Three cases of stage 3 maxillary BRONJ are presented. They were diagnosed and surgically treated with sequestrectomy and reconstruction using pedicled BFP and primary mucosal closure at the Oral and Maxillofacial Department of University Central Hospital between 2008 and 2009.

 -Case 1

A 62-year-old woman presented in March 2008 with pain and swelling in the left upper molar region. The patient presented history of breast cancer diagnosed in 2000 treated with zoledronate (Zometa®, 4 mg IV once every 1 month) for 24 months, from 2005 to 2007. She underwent first upper molar extraction in another centre in January 2008. 

**Figure 1:**
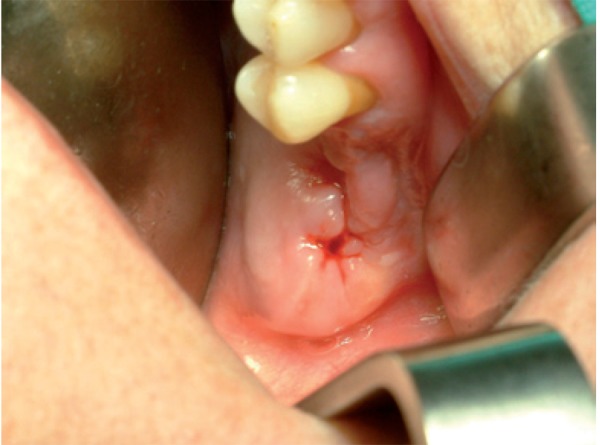
Photograph showing swelling area in upper left maxilla without bone exposure.

**Figure 2:**
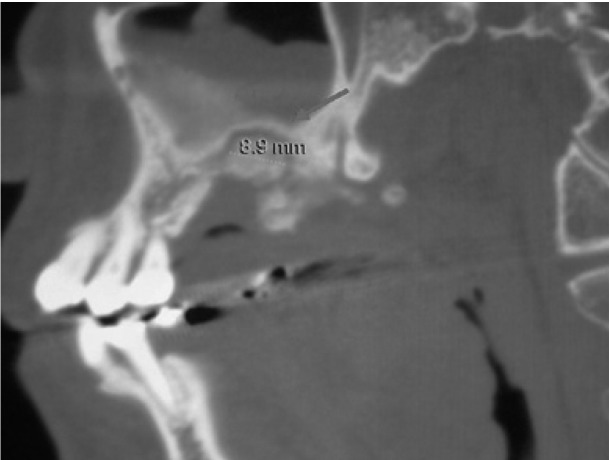
CT image demonstrating bone sequestrum of 8.9 mm diameter in the affected area (red arrow).

Intraoral examination revealed swelling and infection at the same site of molar extraction, but any bone exposure was observed (Fig. 1). Panoramic radiograph was not conclusive, but Computed Tomography (CT) images revealed an 8.9 mm bone sequestrum in the affected area (Fig. 2).

A diagnosis of BRONJ stage 3 was made. We applied medical therapy: amoxicillin (4 gr/day) and clavulanate (250 mg/day) was started and continued for 15 days. Mouth-washes with chlorexidine and hydrogen peroxide were also prescribed. Then, sequestrectomy and bone debridement was performed under general anaesthesia. 

 Surgical technique

**Figure 3:**
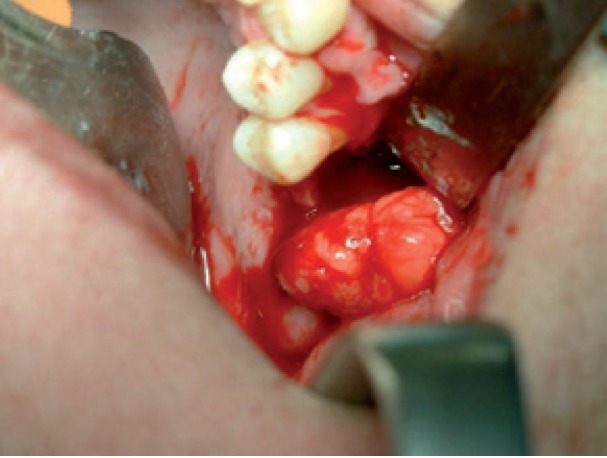
Surgical approach after bone debridement using pedicled BFP and transferring into maxillary defect.

**Figure 4:**
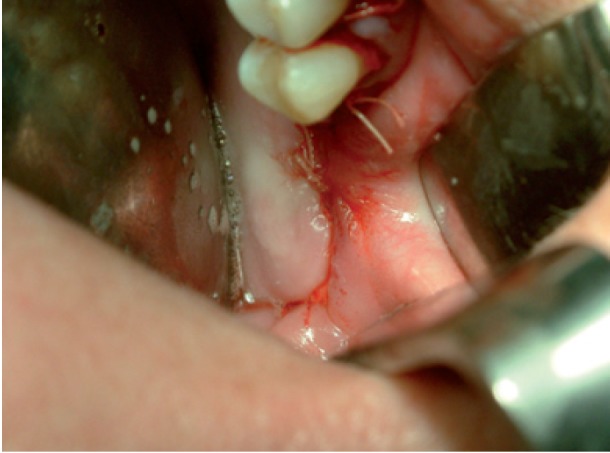
Photograph showing suture of the mucosa over BFP without tension.

Intraoperatively, an incision was made in the superior vestibular sulcus at about 10 mm from the inserted gingiva beginning at the level of the upper second molar, exposing the maxillary periosteum and the BFP. The fat pad was delivered into the mouth by pulling it by blunt dissection, rotated and transferred onto the maxillary defect (Fig. 3). The overlying mucosa was sutured over the BFP without tension (Fig. 4). 

**Figure 5:**
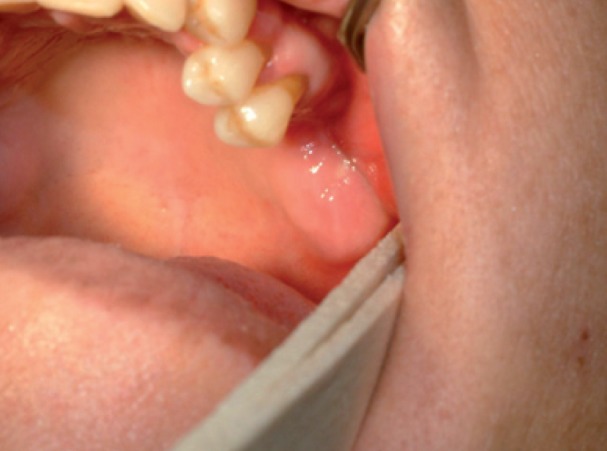
Clinical image six months after surgery.

Postoperative healing was uneventful. No dehiscence, infection, or necrosis was observed (Fig. 5). No oroantral communication was observed. No new oral lesions were observed after 20-months carefully follow-up. 

 -Case 2

**Figure 6:**
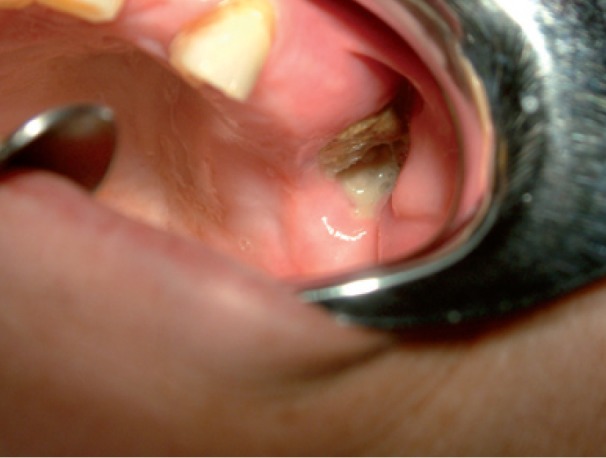
Clinical image revealing alveolar bone exposure of the edentulous posterior maxilla with a purulent discharge.

**Figure 7:**
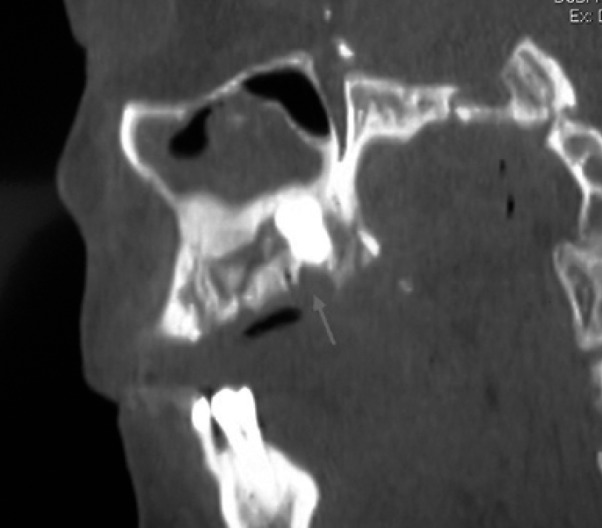
Sagital CT image demonstrating bone sequestrum and third molar inclusion (red arrow).

**Figure 8:**
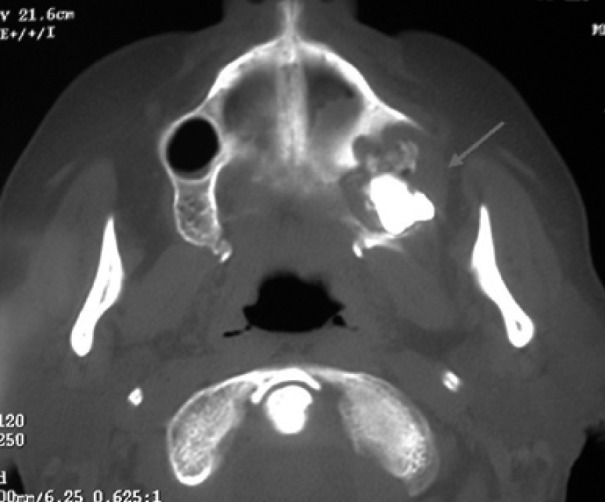
Axial CT image showing large sequestrum around third molar included (red arrow).

A 64-year-old woman was referred in January 2009 by her oncologist for diagnosis of a painful oral lesion. Her past medical history included breast cancer since 1999, treated monthly with intravenous zoledronate infusions, at a dose of 4 mg, from 2006 to 2008. Three years after initiating therapy with zoledronate, the patient complained of discomfort and drainage in the edentulous left upper molar area. The patient did not report previous tooth extraction, and found it difficult to eat, speak, and perform oral hygiene. Intraoral examination revealed that the alveolar bone of the edentulous posterior maxilla was exposed with a purulent discharge (Fig. 6). The surrounding soft tissue was erythematous and edematous. CT images revealed a bone sequestrum and third upper molar impacted in the affected area (Figs. 7,8), and diagnosis of BRONJ stage 3 was made.

**Figure 9:**
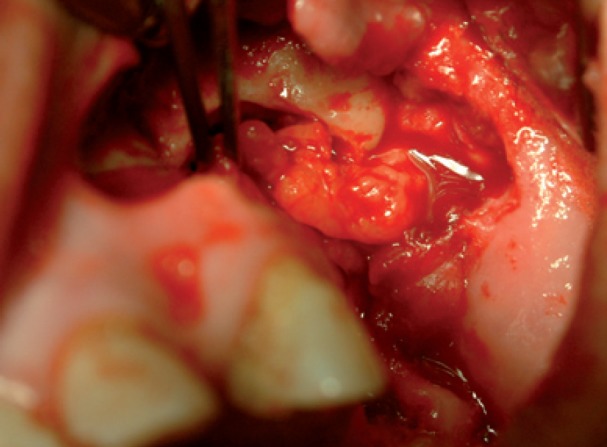
Surgical approach after bone debridement using pedicled BFP into maxillary defect.

**Figure 10:**
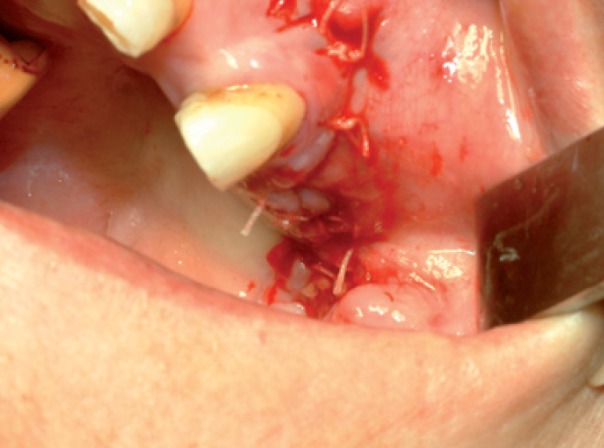
Photograph showing suture of the mucosa over BFP without tension.

Medical therapy with amoxicillin (4 gr/day), clavulanate (250 mg/day) and chlorexidine mouth-washes were started and continued for 15 days. Then, sequestrectomy and third molar extraction was performed under general anaesthesia. Intraoperatively, reconstruction using BFP was performed as described in Case 1 (Figs. 9,10).

Postoperative healing was uneventful with complete healing one month after surgery. No new oral lesions were observed after 10-months carefully follow-up. 

 -Case 3

A 54-year-old woman presented in April 2005 with pain and swelling in the posterior right side of the maxilla. The patient reported first right maxillary molar extraction three months before. Clinical inspection showed a 2mm fenestration that exposed a whitish necrotic centre in the affected area. Computed tomography (CT) showed right maxillary bone sequestrum. Eighteen months before she had had multiple myeloma (isotype IgG) that had been treated with zoledronate (Zometa® 4mg given intravenously every 3 weeks). 

A diagnosis of BRONJ stage 3 of the jaws was made. In August 2005 zoledronate was discontinued in consultation with her oncologist. It was performed sequestrectomy and bone debridement under general anaesthesia. The overlying mucosa was sutured over the defect without reconstruction with BFP. There had been oroantral communication after sequestrectomy in the early postoperative period, closing completely at 8-week follow-up. Treatment with zoledronate was not restarted.

She was followed-up annually. In October 2007 she developed pathological fractures of the bilateral humerus and right femur that were treated with an intramedullary nail that was locked with proximal and distal interlocking screws. Then, the oncologist restarted therapy with zoledronate (Zometa® 4mg given intravenously every month).

**Figure 11:**
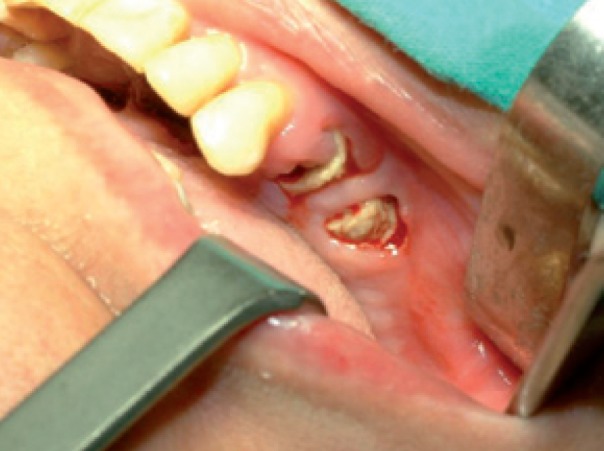
Photograph showing exposed devitalized bone in the upper left edentulous maxillary area.

**Figure 12:**
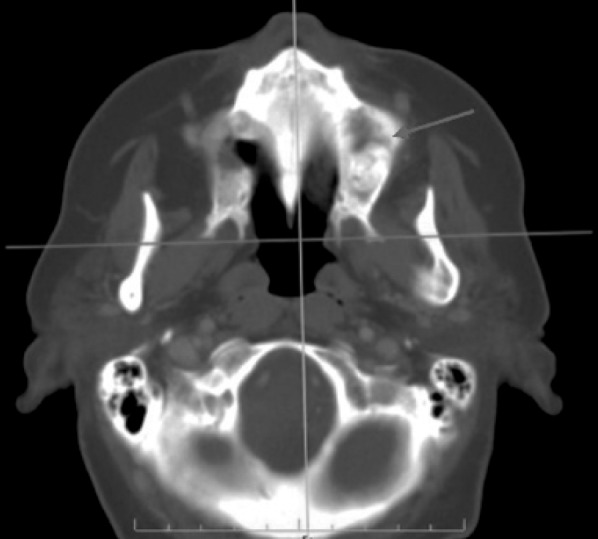
CT image demonstrating a large bone sequestrum in the affected area (red arrow).

**Figure 13:**
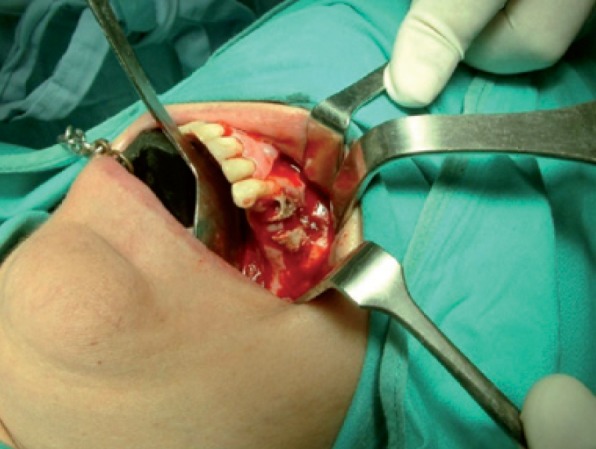
Photograph of the surgical approach showing bone sequestrum before debridement.

**Figure 14:**
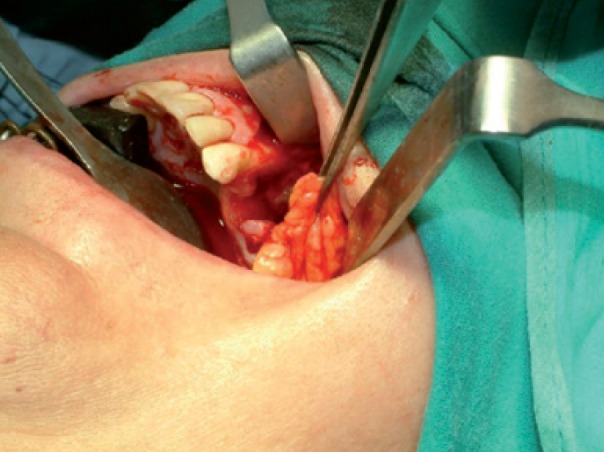
The fat pad was delivered into the mouth by pulling it by blunt dissection, rotated and transferred onto the maxillary defect.

**Figure 15:**
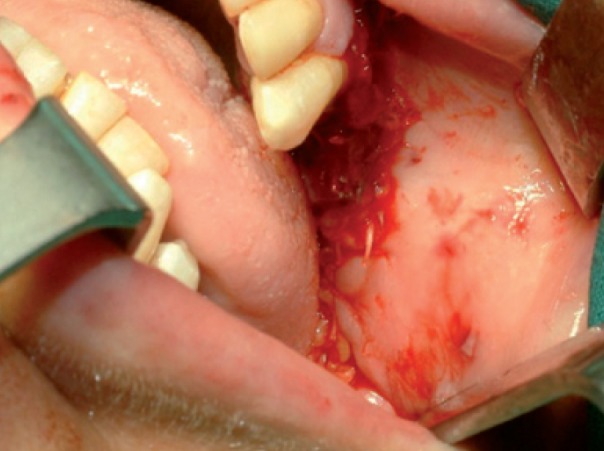
Photograph showing final suture of the mucosa over BFP without tension.

The patient was carefully followed-up and in July 2009 referred pain and swelling in the upper left edentulous maxillary area. The clinical finding was an area of ulcerated mucosa and exposed devitalized bone (Fig. 11) and CT images revealed a large bone sequestrum (Fig. 12). Then, the patient underwent surgical bone debridement and sequestrectomy under general anaesthesia, and reconstruction using BFP was performed as described in Case 1 (Figs. 13, 14,15).

After this second surgery, no oroantral communication was observed and the patient showed acceptable healing and resolution of their disease 6 weeks after surgery. The patient died 7 months after surgery of her systemic disease.

## Discussion

The successful application of BFP has been documented for reconstruction of the palatal region, buccal mucosa, closure of oronasal fistulas, coverage of the surface of bone grafts, and reconstruction after post-traumatic or oncologic defects in the maxillary region (9-12). Hao (9) reported that the ideal defects to be reconstructed with a BFP are the maxillary defects due to their close anatomical location. However, it can be applied in areas ranging from the mouth angle to the retromolar trigone and palate.

The BFP separates the masticatory muscles. The fat tissue is of the syssarcosis type, not subject to lipid metabolism, resembling periorbital fat tissue and can be easily distinguished from subcutaneous fat tissue. The BFP consists of an encapsulated body with 4 extensions: buccal, pterygoid, superficial, and deep temporal. The blood supply to the BFP derives from the buccal and deep temporal branches of the maxillary artery, from the transverse facial branch of the superficial temporal artery, and from some small branches of the facial artery (13).

The perceived limitations of surgical therapy for BRONJ have been discussed extensively in the literature, and recommendations have largely been made to offer conservative therapy to patients, with aggressive surgery offered only to stage 3 patients refractary to conservative management (6,14). Otherwise, many cases do not respond to conservative management and the infection and bone destruction are progressive. Williamson (8) have proposed surgical management of BRONJ included debridement of all necrotic bone, surgical smoothing of the irregular bony areas, tension free primary closure of the wound site with pre and postoperative antibiotics for recalcitrant cases. Carlson et al. (7) described that healing is particularly predictable after resection of the maxilla and mandible in patients in whom BRONJ develops related to a parenteral or an oral bisphosphonate medication. Thus, more aggressive surgery may represent an effective management of stage 3 BRONJ patients. 

When BRONJ area or bone sequestrum is localized in upper maxilla and surgery treatment is planned, OAC must be considered as a potential complication. We postulate that managing the site with BFP and primary closure may ensure a sufficient blood supply and adequate protection for an effective bone-healing response to occur. The BFP must be gently brought into the surgical defect while maintaining the vascular pedicle and it must not be sutured under tension. This flap provides a rich vascularization in the BRONJ site, a mechanic protection and an abundant source of adipose-derived adult stem cells. Those cells have been widely used in bone engineering due their capacity to differentiate along multiple mesodermal lineage pathways and form osteoid matrix in vivo (15).

## Conclusions

In summary, the BFP is a simple procedure, widely applicable with low incidence of failure and minimal donor site morbidity that may be recommended when surgery in upper maxilla in BRONJ patients is planned. This technique could reduce or avoid postsurgical OAC and promote bone healing in the affected area.
